# American Academy of Dental Science

**Published:** 1871-11

**Authors:** E. N. Harris

**Affiliations:** Corresponding Sec'y.


### ARTICLE III.

### *American Academy of Dental Science.*

The fourth annual meeting of the American Academy of Dental Science, was held in Boston, at Christian Union Hall, 300 Washington St., at 10 o'clock, A. M., September 25th, 1871; the President, Dr. Daniel Harwood, in the chair.

The usual reports were read and accepted, showing the Society to be in a flourishing condition. The Censors reported the names of several gentlemen who were elected members of the Academy.

The following officers were chosen for the ensuing year: *President*, Daniel Harwood, M. D.; *Vice-President*, E. T. Wilson, M. D.; *Recording Secretary*, L. D. Shepard, D. D. S.; *Corresponding Secretary*, E. N. Harris, D. D. S.; *Treasurer*, E. G. Tucker, M. D.; *Librarian*, John Clough, M. D.

*Board of Censors*—E. G. Tucker, M. D.; J. L. Williams, M. D.; W. W. Codman, M. D.

At one o'clock the annual address was delivered by Prof. J. H. McQuillen, M. D. D. D. S., of Philadelphia. His subject was "Dental Education." The address was a very fine production, and was delivered in an earnest and eloquent manner, eliciting the interested attention and hearty approval of the assembly.

At its conclusion it was voted "That the thanks of the Academy be tendered to Dr. McQuillen, of Philadelphia, for his very able address, and that a copy be requested for publication and preservation in our archives."

Essays were then read by Dr. James McManus, of Hartford, on "Dental Operations;" Dr. E. N. Harris, of Boston, on "Causes of Caries of the Teeth, and General Means for Preserving the Teeth;" and Dr. L. D. Shepard, of Boston, on "The Merits of the Old and New Methods of Practice."

A vote of thanks was presented to the essayists.

At 4 o'clock A. M., the Academy adjourned to the Parker House, where they partook of an excellent dinner, and enjoyed therewith many pleasant and appropriate speeches from those present.

After the dinner, the members were invited to the rooms of Prof. McQuillen, who exhibited some interesting and valuable microscopic preparations.

The Academy holds regular monthly meetings in Boston, which are well attended, and the time pleasantly and profitably occupied in discussions, reading of papers, presentations of specimens of morbid anatomy, operative and mechanical dentistry, &c.

E. N. Harris, D. D. S., Corresponding Sec'y.

---

